# Excision of the Knee-Joint

**Published:** 1899-11-25

**Authors:** 


### EXCISION OF THE KNEE-JOINT.

The history of excision of the knee-joint is a curious one. It seems to have been originally performed by Parke in 1781, but only very occasionally repeated until its revival by Ferguson in 1850, when it rapidly became popular and was performed in a large number of cases, the operation being done, at any rate at first, as a substitute for amputation, and mostly in cases of tuberculous disease of the knee. When, however, about 1870, the introduction of aseptic methods enabled the operation to be done with scarcely any appreciable risk, when primary union could be reckoned upon, and when patients were able to use the limb in the course of three or four months, the conditions were completely changed, and the operation, which had been used chiefly as an alternate for a severe mutilation, became a procedure which it was justifiable to undertake in the early stages of tuberculous disease, with the object of removing structures involved in an infection which might, if not eradicated, spread to other parts. The ultimate results of the operation in children were, however, so unfavourable, partly from interference with growth, and partly from yielding of the uniting material, that the operation to a large extent fell into disuse, except in cases in which deformity could not otherwise be corrected. Further experience, however, has shown that there are several conditions in which excision of the knee yields admirable results.

In a clinical lecture delivered at St. Bartholomew's Hospital, Mr. Howard Marsh[1] says that the cases in which excision is advisable are limited, with few exceptions, to adults and adolescents in whom the growth of the limb is complete, or nearly so, and in whom the bones have acquired so firm a texture that after the operation they readily undergo an unyielding synostosis. Such cases may be arranged in the following groups:—

Group I.: Cases in which the joint has become fixed in a position of deformity, the majority of such cases being due to tuberculosis, while some arise from septicæmic, gonorrhœal, and other affections.

Group II.: Cases in which the joint is incapacitated by incomplete fibrous ankylosis, resulting usually either from tuberculosis or septicæmia which has lasted a long time and has to a large extent destroyed the structures of the joint.

Group III.: Cases in which, by a process regarding which very little is at present known, a variable number of joints, one after another, become inflamed, slightly over warm, and somewhat painful, and then, in the course of a few months, fixed by complete bony ankylosis in a position of flexion. In such cases, when the knees are the only joints affected in the lower extremities, rectification of position by excision may enable the patient to become to some extent active again.

Group IV.: Cases in which, as the result of osteoarthritis, the knee-joint has undergone extensive structural change, resulting in either a loose flail-like condition, or in fixation at such an angle as to render it useless. Of course in such cases the condition of the patient or the fact that the disease has affected many large joints may contraindicate any operation. But when such conditions are absent operation may sometimes be attended with very great benefit. For whatever condition excision of the knee is performed it is well to remember that the good progress of the case depends upon maintaining the close apposition of the bones after the operation. The method which Mr. Marsh finds most useful for this purpose is a combination of Gant's splint and the use of sterilised steel pins. The limb is first placed on the back splint, the tibia being raised to the level of the femur by additional pads placed under the calf, and then the steel pins are inserted, parallel to each other, one through the internal tuberosity of the tibia and the internal condyle of the femur, and the other through the external tuberosity and external condyle. The outside splint is then applied. The pins are removed between the tenth and the fifteenth day. This is easily done by means of the clamp handles, by which they are originally introduced.

[1] Lancet, Nov. 18.

